# Cloning, expression, tertiary structure, activity characteristics, design of inhibitor, hydrolysis and inhibition mechanisms analysis of carp adipose triglyceride lipase (ATGL)

**DOI:** 10.1016/j.fochms.2026.100460

**Published:** 2026-09-30

**Authors:** Qinghai Wang, Haixia Zhong, Yanhua Jia, Anhu Wang, Zhiguang Chen

**Affiliations:** a Xichang University, College of Agricultural Sciences, Panxi Crop Improvement Key Laboratory of Sichuan Province, Xichang, Sichuan Province 615000, China; b Xichang University, College of Resources and Environment, Sichuan Province, Xichang, Sichuan Province 615000, China

**Keywords:** Carp, ATGL, Activity characteristics, Tertiary structure, Hydrolysis mechanism

## Abstract

Lipid hydrolysis catalyzed by endogenous enzymes is a major factor reducing the quality of meat products. Adipose triglyceride lipase (ATGL) initiates triacylglycerol hydrolysis and is therefore a key enzyme in this process. In this study, the N-terminal domain of carp ATGL was cloned. Recombinant ATGL was successfully expressed and purified, and its activity was characterized at different temperatures and pH values. The predicted tertiary structure of ATGL was analyzed. Carp ATGL is a monomeric protein composed of nine α-helices and seven β-strands, with a radius of gyration (Rg) of 1.84 nm. Its active center is a catalytic dyad (Ser47-Asp166) located at the bottom of an active groove that is predominantly hydrophobic. The active groove is surrounded by three flexible and hydrophobic loops, which are involved in binding hydrophobic lipids. The hydrolysis mechanism of ATGL was then elucidated. Finally, an inhibitor of ATGL was designed: a small peptide consisting of hydrophobic essential amino acids (Lys-Leu-Ile-Met-Val). After the addition of the small peptide, lipid hydrolysis and oxidation in carp surimi during storage were effectively controlled. And this peptide can be misrecognized by the hydrophobic active groove of ATGL and forms a steric hindrance, thereby preventing triacylglycerol binding.

## Highlights

• Carp ATGL was cloned, expressed and purified.
• The activity characteristics, tertiary structure and hydrolysis mechanism of carp ATGL were analyzed.
• An inhibitor of ATGL was designed and the inhibition mechanism were analyzed.
• An ATGL inhibitor of essential amino acids is a nutritional fortifier, promising as a carp meat product additive.
• ATGL misrecognizes the inhibitor in its hydrophobic active groove, causing steric hindrance that blocks triglyceride binding

## 1 Introduction

Carp (*Cyprinus carpio*) is an economically important freshwater fish, with an aquaculture production of approximately 3 million tonnes in 2025 in China (Chinese Fisheries Statistical Yearbook, 2025). Owing to its excellent meat quality and high nutritional value, carp is highly favored by both consumers and producers (Li et al., 2023). However, compared with other meats, fish is more prone to spoilage during processing or storage due to abundant endogenous enzymes, high muscle moisture content, delicate muscle tissue, and diverse bacteria attached to the digestive tract or gills (Bade et al., 2025; Cheng et al., 2025; Sobczak et al., 2020)..

Among these factors, lipid decomposition caused by endogenous enzymes is one of the most important contributors to fish spoilage (Zhang et al., 2024a). On one hand, the breakdown of lipids produces small-molecule compounds such as alcohols, aldehydes, and ketones, which seriously affect fish quality (Liu, 2013). On the other hand, excessive lipid decomposition accelerates protein degradation, leading to softening of fish meat or surimi gel (an important processed product of fish) (Eymard et al., 2015).

Lipid decomposition includes both oxidation and hydrolysis, which can promote each other. Lipid oxidation comprises autoxidation, photosensitized oxidation, and enzymatic oxidation by lipoxygenases (LOXs) (Han et al., 2020). Lipid hydrolysis is mainly mediated by three intracellular enzymes: adipose triglyceride lipase (ATGL) (Cerk et al., 2018), hormone-sensitive lipase (HSL) (Recazens et al., 2021), and monoglyceride lipase (MGL) (Zanfirescu et al., 2021) . ATGL is primarily responsible for hydrolyzing triacylglycerols; HSL primarily hydrolyzes diacylglycerols; and MGL primarily hydrolyzes monoacylglycerols (Cerk et al., 2018; Recazens et al., 2021; Zanfirescu et al., 2021).

ATGL hydrolyzes triacylglycerols into diacylglycerols and free fatty acids. It is predominantly expressed in adipose tissue and heart (Cerk et al., 2018; Pulinilkunnil et al., 2014; Schreiber et al., 2019), and is also expressed in other tissues such as liver, kidney, pancreas, and muscle (Li et al., 2025; Marzouk et al., 2025; Tang et al., 2013; Zheng et al., 2024). Regarding fish growth and flesh quality, studies have shown that the growth rate, biochemical parameters, and immune indices of tilapia are associated with ATGL expression (Marzouk et al., 2025). Furthermore, when the ATGL inhibitor Atglistatin was supplemented in the diet of zebrafish, severe fat deposition occurred, accompanied by reduced oxygen consumption and fatty acid β-oxidation levels, as well as aggravated oxidative stress and enhanced inflammatory responses (Han et al., 2021). ATGL catalyzes the hydrolysis of triacylglycerols and is considered a rate-limiting enzyme in the lipolytic cascade; lipid hydrolysis can further promote lipid oxidation, and both lipolysis and oxidation are key factors contributing to the deterioration of fish flesh quality. Moreover, residual ATGL activity has been detected in muscle tissues (Alsted et al., 2009; Zheng et al., 2024). Therefore, it is theoretically reasonable to speculate that endogenous ATGL is closely related to changes in fish flesh quality.

In this study, carp ATGL was cloned and prokaryotically expressed. Subsequently, its enzymatic activity characteristics, predicted tertiary structure, and hydrolysis mechanism were analyzed. Finally, an inhibitor of ATGL was designed and its inhibition mechanism was studied. These results provide an important reference for basic research on lipid hydrolases and also offer a basis for controlling carp meat quality during processing.

## 2 Materials and methods

### 2.1 Cloning and expression of ATGL

RNA was extracted from the dorsal muscle of carp and characterized by agarose gel electrophoresis. The extracted RNA was reverse-transcribed into cDNA using a reverse transcription kit (GeneCreate Bioengineering Co., Ltd., Wuhan, China). PCR amplification was performed using the cDNA as a template with the following primers: 5′-atgtttcccctcgactctccgt-3′ and 5′-taacagaccgttcttcttcag-3′. Primers were designed with reference to the sequence A0A9J7Z9S4_CYPCA from the Universal Protein Resource (UniProt) database. The PCR products were sequenced by a biotechnology company (GeneCreate Bioengineering Co., Ltd., Wuhan, China) to verify their correctness. The sequence was analyzed by using DNAMAN 9.0. It should be noted that the full-length ATGL consists of 1491 bp encoding 497 amino acids. In this study, only the N-terminal domain harboring the active region was cloned; this domain is 768 bp in length and encodes 256 amino acids. Following initial cloning and sequencing, the sequence encoding the ATGL N-terminal domain was re-synthesized based on the sequencing results for construction of a recombinant expression system. For this purpose, *Nde*I and *Xho*I restriction sites were incorporated during gene synthesis, and the protective bases GGAATTC and CCG were added at the 5′ and 3′ ends, respectively, to facilitate efficient restriction digestion. The resulting expression vector, pET-30a-ATGL-N, was transformed into competent *E. coli* BL21(DE3) cells for inducible expression.

The harvested cells were resuspended in phosphate-buffered saline (PBS) and centrifuged to remove the residual culture medium. The cell pellet was then resuspended in PBS and disrupted by ultrasonication on ice (300 W, 3 s on and 6 s off) until the suspension became translucent. The lysate was centrifuged at 10,000 ×*g* for 10 min at 4 °C, and the supernatant was discarded. The resulting inclusion body pellet was sequentially washed with the following buffers: first with 2 M urea buffer (50 mM Tris-HCl, pH 8.0, 100 mM NaCl, 2 M urea, 0.5% Triton X-100), and then with 4 M urea buffer (50 mM Tris-HCl, pH 8.0, 100 mM NaCl, 4 M urea). After each washing step, the suspension was centrifuged at 12,000 ×*g* for 15 min at 4 °C, and the supernatant was discarded. The washed inclusion bodies were dissolved in solubilization buffer (50 mM Tris-HCl, pH 8.0, 100 mM NaCl, 8 M urea, 10 mM β-mercaptoethanol). The sample was loaded onto a Ni-NTA column pre-equilibrated with binding buffer (20 mM Tris-HCl, 0.5 M NaCl, 20 mM imidazole, pH 8.0) at a flow rate of 0.5 mL/min. After loading, the column was washed with wash buffer containing 40 mM imidazole to remove non-specifically bound proteins. The target protein was then eluted with elution buffer containing 250 mM imidazole at a flow rate of 1 mL/min, and the elution peak was collected. The purified protein was diluted to a final concentration of 0.05 mg/mL using the solubilization buffer, and then refolded by stepwise dialysis at 4 °C against refolding buffer (20 mM Tris-HCl, pH 8.0, 100 mM NaCl, 0.5 M L-arginine, 5% glycerol) containing decreasing concentrations of urea (6, 4, 2, 1, and 0 M). The sample was dialyzed for approximately 8 h at each urea concentration, with two final changes of urea-free buffer.

### 2.2 Activity characterization of ATGL

The activity of ATGL was measured using a highly sensitive liquid scintillation counter according to Nagy et al. and Schweiger et al (Nagy et al., 2014; Schweiger et al., 2014) The substrate consisted of 0.33 mM triolein, 0.45 mM phosphatidylcholine/phosphatidylinositol (3:1), and 0.5% defatted bovine serum albumin, with [9,10-^3^H]triolein as the radiotracer. The concentration of purified recombinant ATGL was adjusted to 1 mg/mL by dialysis and concentration. For the reaction, 100 μL of substrate was added to 100 μL of sample, and the reaction was carried out either at pH 3–11 (25 °C) or at 10–50 °C (pH 7) for 30 min. The reaction was terminated by adding 3.25 mL of methanol/chloroform/heptane (10:9:7) and 1 mL of 0.1 M potassium carbonate, 0.1 M boric acid (pH 10.5). After centrifugation at 1000 ×*g* for 15 min, the supernatant was taken for liquid scintillation counting.

ATGL activity was calculated using the following equation: Enzyme activity (nmol FFA/mg ATGL·h) = ΔDPM/(SA × m × t). where ΔDPM = DPMsample − DPMcontrol. The control represents the background measured in the absence of ATGL. In this equation, “m” is the total amount of enzyme protein added (in mg), “t” is the reaction time (in h), and “SA” is the specific radioactivity of the substrate (in DPM/nmol fatty acid equivalent), which was determined to be 6168 under the present assay conditions. All experiments were performed in triplicate.

Specifically, the ATGL activity assay was slightly modified to evaluate the inhibitory effect of the small peptide on ATGL. Synthetic peptide powder (2–20 μg) was added to 100 μL of substrate solution; to ensure more accurate weighing, the mixture was scaled up by a factor of 100 and thoroughly mixed. The final peptide concentrations in the reaction were 0.01, 0.025, 0.05, 0.075, and 0.1 mg/mL. ATGL activity in the presence of the peptide was then measured according to the ATGL activity assay protocol (pH 7, 25 °C). The inhibition rate (%) was calculated as (ATGL activity with peptide / ATGL activity without peptide) × 100% (pH 7, 25 °C). Each concentration gradient was tested in triplicate.

### 2.3 Synthesis and characterization of the inhibitor

The small peptide was chemically synthesized by Changzhou Mukai Biotechnology Co. Its molecular weight and purity were analyzed by mass spectrometry (MS; Agilent 6125B, Agilent Technologies, USA) and high-performance liquid chromatography (HPLC; Agilent 1290 Infinity III, Agilent Technologies, USA), respectively. HPLC conditions: column, 4.6 × 250 mm, Sinochrom ODS-BP 5 μm; mobile phases, 0.1% trifluoroacetic acid in 100% acetonitrile and 0.1% trifluoroacetic acid in 100% water; flow rate, 1.0 mL/min; detection wavelength, 220 nm. MS conditions: electrospray ionization (ESI) probe; probe bias, 4.5 kV; detector, 1.5 kV; nebulizer gas flow rate, 1.5 L/min; T. flow rate, 0.2 mL/min; mobile phase, 50% acetonitrile; block temperature, 200 °C; CDL temperature, 250 °C.

### 2.4 Molecular docking

Based on the sequencing results, the tertiary structure model of ATGL was constructed using the SWISS-MODEL homology modeling tool. The structural models of a small peptide and triacylglycerol were built with PyMOL. The triacylglycerol molecule was trioctanoin (each fatty acid chain contained 8 carbon atoms, with no double bonds and a degree of unsaturation of zero). Molecular docking of ATGL with triacylglycerol or the small peptide was performed using Discovery Studio software according to our previous study (Zhong et al., 2024a). Water molecules in the PDB file were removed, and hydrogen atoms were added. The models were then minimized using a smart minimizer algorithm. Docking results were visualized using VMD.

### 2.5 Molecular dynamics simulation

Molecular dynamics simulations of the ATGL–triacylglycerol and ATGL–small peptide complexes obtained from docking were performed with three parallel simulations per complex. All simulations were performed using an all-atom model. Parameter settings were based on our previous study (Zhong et al., 2024b). Langevin and Berendsen barostats were used to control temperature and pressure, respectively. All simulations were performed using Amber software with the Glycam06, ff14SB, and TIP5P force fields in the NPT ensemble for 100 ns, following 2 ns equilibration in the NVT ensemble and 2 ns in the NPT ensemble. A cutoff distance of 1 nm, periodic boundary conditions, and a leap-frog algorithm were applied.

Simulation snapshots were extracted every 0.01 ns. The radius of gyration (Rg), root-mean-square fluctuation (RMSF), root-mean-square deviation (RMSD), solvent-accessible surface area (SASA), binding free energy, hydrogen bonds, and atomic distances were calculated. Binding free energies were computed using the MM-PBSA method. SASA was determined using the linear combination of pairwise overlap algorithm according to the Weiser definition. Hydrogen bonds were considered present when the maximum distance was ≤0.35 nm.

### 2.6 TBARS value and acid value

50 g of carp flesh sample (approximately 70% moisture) was weighed, and 25 mL of 0.4 mg/mL small peptide solution was added (the control group received 25 mL of distilled water). After homogenization and thorough mixing, the mixture was divided into three portions. After storage at 4 °C for 5 days, TBARS values and acid values were measured.

The effect of the small peptide on the TBARS value of carp flesh was measured according to GB 5009.181–2016. A 3 g flesh sample was weighed, homogenized and extracted with 7.5% trichloroacetic acid-0.1% EDTA, and then centrifuged and filtered. The filtrate was mixed with 0.02 mol/L TBA, heated in a 90 °C water bath for 30 min, cooled, and the absorbance was measured at 532 nm. Quantification was performed using a malondialdehyde standard curve. Each experiment was performed in triplicate. TBARS value (mg MDA/kg) = (C × V × 1000)/(m × 1000). C is the MDA concentration in the sample solution obtained from the standard curve; V is the final volume of the sample solution; and m is the sample mass.

The effect of the small peptide on the acid value of carp flesh was measured according to GB 5009.229–2016. A 3 g flesh sample was weighed, and lipids were extracted with diethyl ether-ethanol (1:2), followed by rotary evaporation to dryness. The residue was dissolved in neutral diethyl ether-ethanol, phenolphthalein was added, and the solution was titrated with 0.1 mol/L KOH ethanol solution until a faint red color persisted for 30 s. Each experiment was performed in triplicate. AV = [(V − V₀) × C × 56.1]/m. In the formula, V is the volume of KOH standard solution consumed by the sample; V₀ is the volume of KOH standard solution consumed in the blank test; C is the concentration of the KOH standard solution; and m is the sample mass.

## 3 Results and discussion

### 3.1 Cloning of carp ATGL

As shown in Fig. S1a, ATGL from various species (human, mouse, pig, and others) contains two domains, with the N-terminal domain being essential for catalytic activity (Kulminskaya & Oberer, 2020). In vitro studies have shown that ATGL remains catalytically active even in the absence of the C-terminal domain (Schreiber et al., 2019).

In this study, the N-terminal domain of carp ATGL was cloned from carp muscle tissue. As shown in Fig. S1b, RNA extracted from muscle tissue displayed three bands (28S, 18S, and 5S). The RNA was reverse-transcribed to cDNA, which was used as a template for ATGL cloning. Agarose gel electrophoresis (Fig. S1c) revealed a single band of approximately 750 bp, matching the expected size of the ATGL N-terminal domain (Auger et al., 2019; Cerk et al., 2018; Kulminskaya & Oberer, 2020). This band was excised, recovered, and sequenced. Sequencing results (Fig. S1d) showed that the carp ATGL N-terminal domain is 768 bp long, encoding 256 amino acids with a molecular weight of 27.7 kDa and a pI of 7.98. Sequence similarity between carp ATGL and ATGL from other species was approximately 80%: *Larimichthys crocea* (HQ916211) 87.11%, *Homo sapiens* (AY894804) 78.13%, *Sus scrofa* (EF583921) 76.56%, *Mus musculus* (AY894805) 79.24%, *Bos taurus* (FJ897536) 78.91%, *Anser anser* (HQ914789) 80.47%, and *Coturnix japonica* (EU852336) 80.08% (Fig. S2). ATGL is highly conserved across species. For comparison, the similarity of another lipid hydrolase, hormone-sensitive lipase (HSL), between human and carp is only about 50% (Zhong et al., 2021), whereas ATGL similarity can be as high as ∼80%.

### 3.2 Prokaryotic expression and activity analysis of ATGL

To characterize ATGL activity, ATGL was prokaryotically expressed in *E. coli*. Based on the sequencing results, the ATGL sequence was synthesized and ligated into the PET-30a vector, and the recombinant plasmid was transformed into *E. coli* BL21. As shown in Fig. 1a, a strong protein band appeared at approximately 27 kDa after induction, indicating successful expression of ATGL. The recombinant protein was present in the lysis precipitate, i.e., it formed inclusion bodies. After washing, the sample was purified by nickel-affinity chromatography. Following dialysis and concentration, a single band was observed on SDS-PAGE (Fig. 1a,b), indicating successful purification of recombinant ATGL. The relatively large contaminant protein peak observed during nickel affinity chromatography may be attributed to factors such as excessive sample loading or an overly high flow rate. Studies have shown that the ATGL N-terminal domain in its monomeric form possesses a complete and preassembled catalytic center (Cerk et al., 2018; Kulminskaya & Oberer, 2020; Schreiber et al., 2019). In the present study, recombinant expression of the N-terminal domain of ATGL further demonstrated that the monomeric form of this domain is catalytically active.

**Fig. 1:**
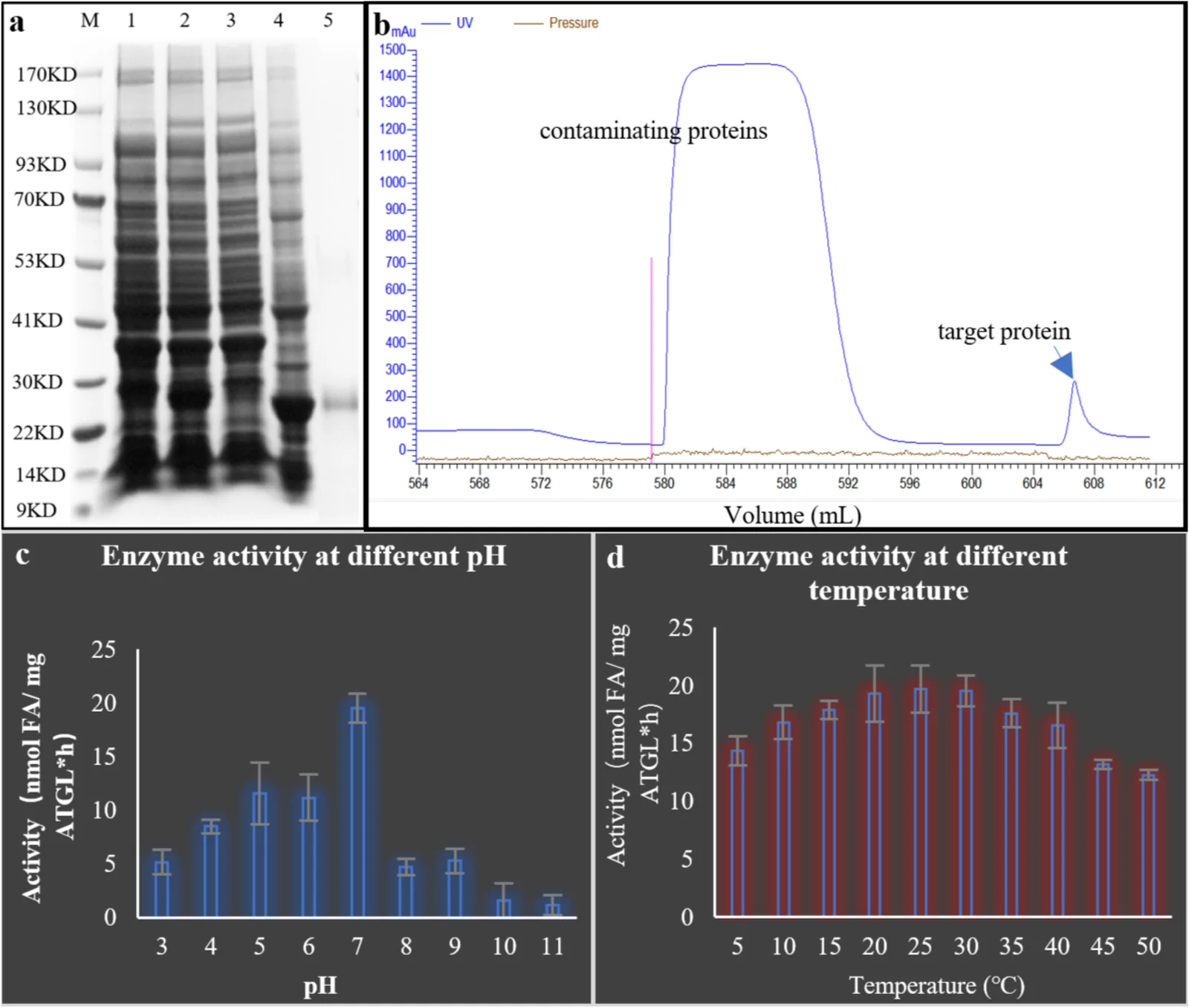
The prokaryotic expression and activity characteristics of carp ATGL. a: SDS PAGE electrophoresis of ATGL induced expression, M: marker, 1: pre-induction, 2: after induction, 3: supernatant, 4: precipitate, 5: purified ATGL. b: Nickel column affinity chromatography of ATGL.; c: ATGL activity at different pH, d: ATGL activity at different temperature.

The activity of ATGL at different pH values and temperatures is shown in Fig. 1c,d. Activity was highest at pH 7, and any increase or decrease in pH reduced activity (Fig. 1c). Alkaline conditions had a stronger inhibitory effect than acidic conditions. Even at pH 3, ATGL retained about 26.4% of its maximal activity. Fresh carp meat has a pH of approximately 7.1. During storage, pH gradually decreases, reaching a minimum of 6.7 on day 4, and then gradually returns to 7.1 by day 10 (Wang et al., 2020). Furthermore, studies found that both the gel strength and water-holding capacity of surimi gel were optimal when the pH was adjusted to 8.0 (Gao et al., 2018). Therefore, in practical applications, adjusting the storage pH to a slightly alkaline condition can not only ensure the quality of surimi gel but also effectively inhibit ATGL activity.

The effect of temperature on ATGL activity was less pronounced than that of pH. Activity changed little between 20 and 35 °C. When the reaction temperature was lowered to 5 °C or raised to 50 °C, ATGL retained about 73.6% and 62.8% of its activity, respectively. This suggests that low-temperature storage alone cannot completely control triacylglycerol hydrolysis in carp meat.

### 3.3 Tertiary structure of ATGL

The tertiary structure of ATGL was characterized by molecular dynamics simulation. A 100-ns simulation was performed (Fig. 2a). The RMSD values increased during the first 10 ns and then stabilized, indicating a well-behaved model without energy anomalies (Hu et al., 2023).

**Fig. 2:**
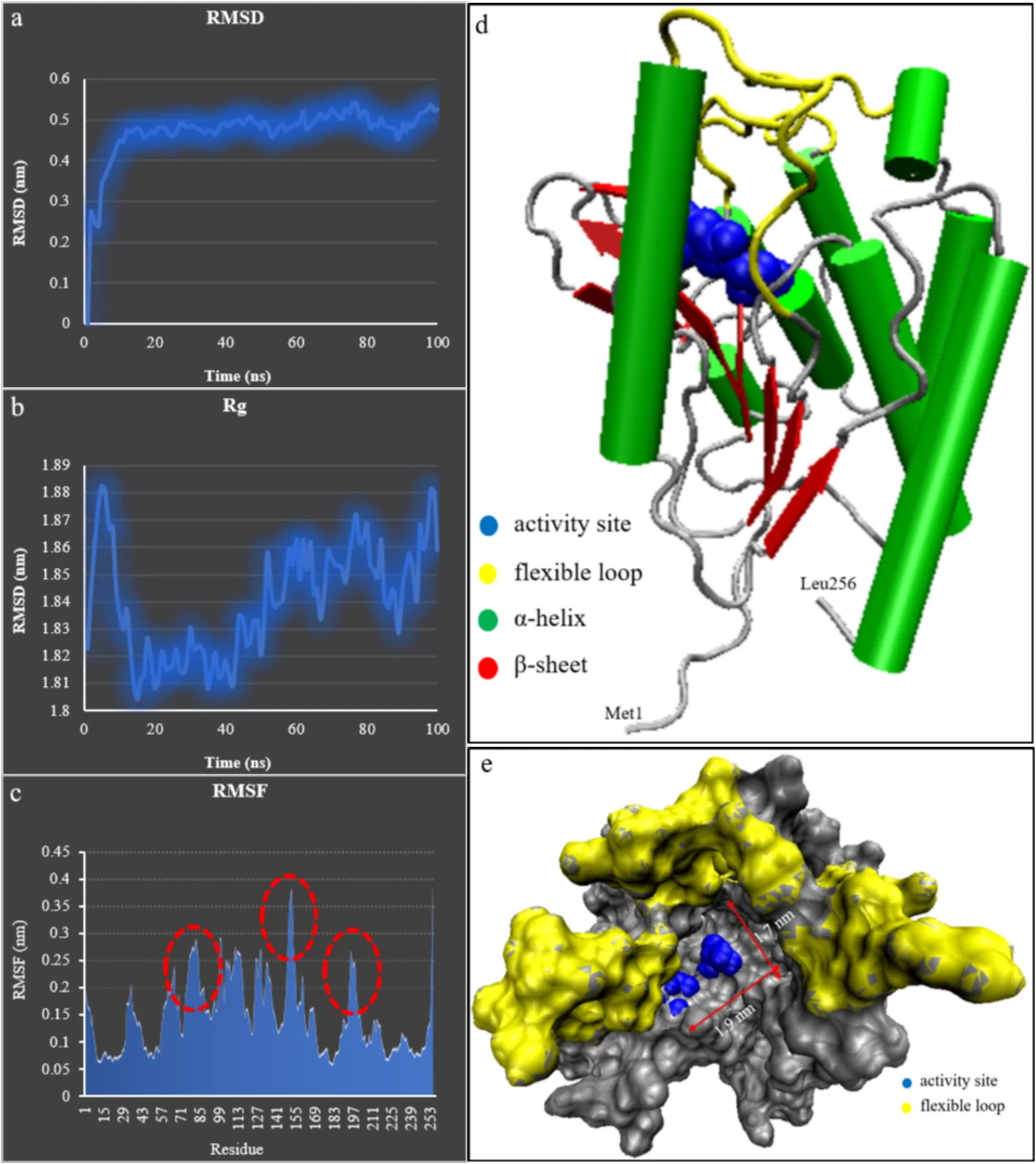
Carp ATGL tertiary structure. a: Rg of ATGL, b: RMSD of ATGL, c: RMSF of ATGL, d and e: Tertiary structure of ATGL.

As shown in Fig. 2b and d, carp ATGL is a monomeric protein with no disulfide bonds and an Rg of approximately 1.84 nm. It consists of nine α-helices and seven β-strands. The seven β-strands are arranged in parallel or antiparallel fashion, and the nine α-helices are distributed around the β-strands. RMSF values (Fig. 2c) were low in β-strands and α-helices, whereas loop structures exhibited higher flexibility. Three regions with high RMSF deserve attention: the loop between α4 and α5 (Ser87-Arg95), the loop between α7 and β5 (Tyr147-Pro156), and the loop between β6 and α8 (Ser188-Asp197). As seen in Fig. 2e, these three highly flexible loops are located above the active center of ATGL and may be closely related to substrate binding.

The active center of ATGL is a catalytic dyad (Ser47-Asp166) located at the bottom of an active groove approximately 1.9 nm long and 1.7 nm wide (Fig. 2f). Hydrophobicity analysis was performed by using Kyte-Doolittle method. As shown in Fig. 3, the analysis revealed that the vast majority of residues at the bottom of the active groove are hydrophobic, including Pro14, Trp15, Asn16, Ile17, Asn46, Arg48, Tyr51, Asn142, Glu143, Glu144, Leu145, Thr165, Gln167, and Gly168. This facilitates binding of hydrophobic fatty acid chains. Most residues on the surface of ATGL are hydrophilic, consistent with a typical protein structure (hydrophilic exterior, hydrophobic interior) (Kamal et al., 2023). Notably, among the three highly flexible loops mentioned above, two are fully hydrophobic (Ser87-Arg95 and Tyr147-Pro156) and the third is partially hydrophobic (Ser188-Asp197). This further supports the involvement of these loops in binding hydrophobic lipid chains.

**Fig. 3:**
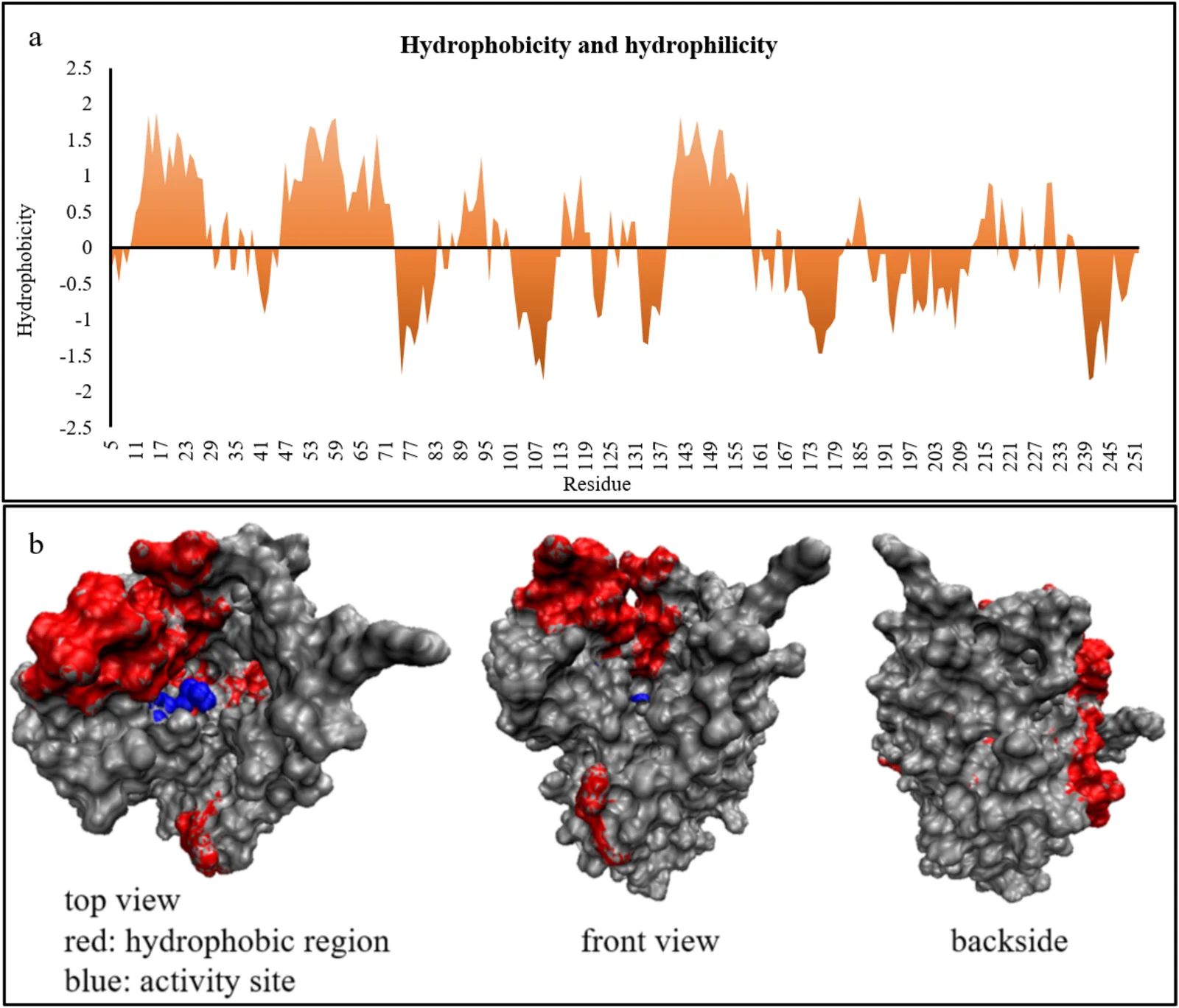
Hydrophilicity analysis of carp ATGL.

### 3.4 Catalytic mechanism of ATGL

The interaction between ATGL and triacylglycerol molecule was analyzed by molecular docking. Interaction sites were identified based on changes in SASA. After complexation with triacylglycerol, SASA decreased significantly at several residues, including Ser47, Leu90, Tyr151, Asp166, and Ile193 (Fig. 4). The interaction sites are located in the active groove and the highly flexible loops. As shown in Fig. 4b, the triacylglycerol molecule is embedded in the active groove, with its ester bond in close contact with the catalytic dyad. The distances between the catalytic-site residues Asp166 and Ser47 and the ester bond of the triacylglycerol molecule were 0.374 nm and 0.401 nm, respectively. As the simulation progresses, the three flexible hydrophobic loops undergo conformational changes and gradually encapsulate the triacylglycerol molecule inside the cavity, ensuring efficient hydrolysis (Fig. 4c).

**Fig. 4:**
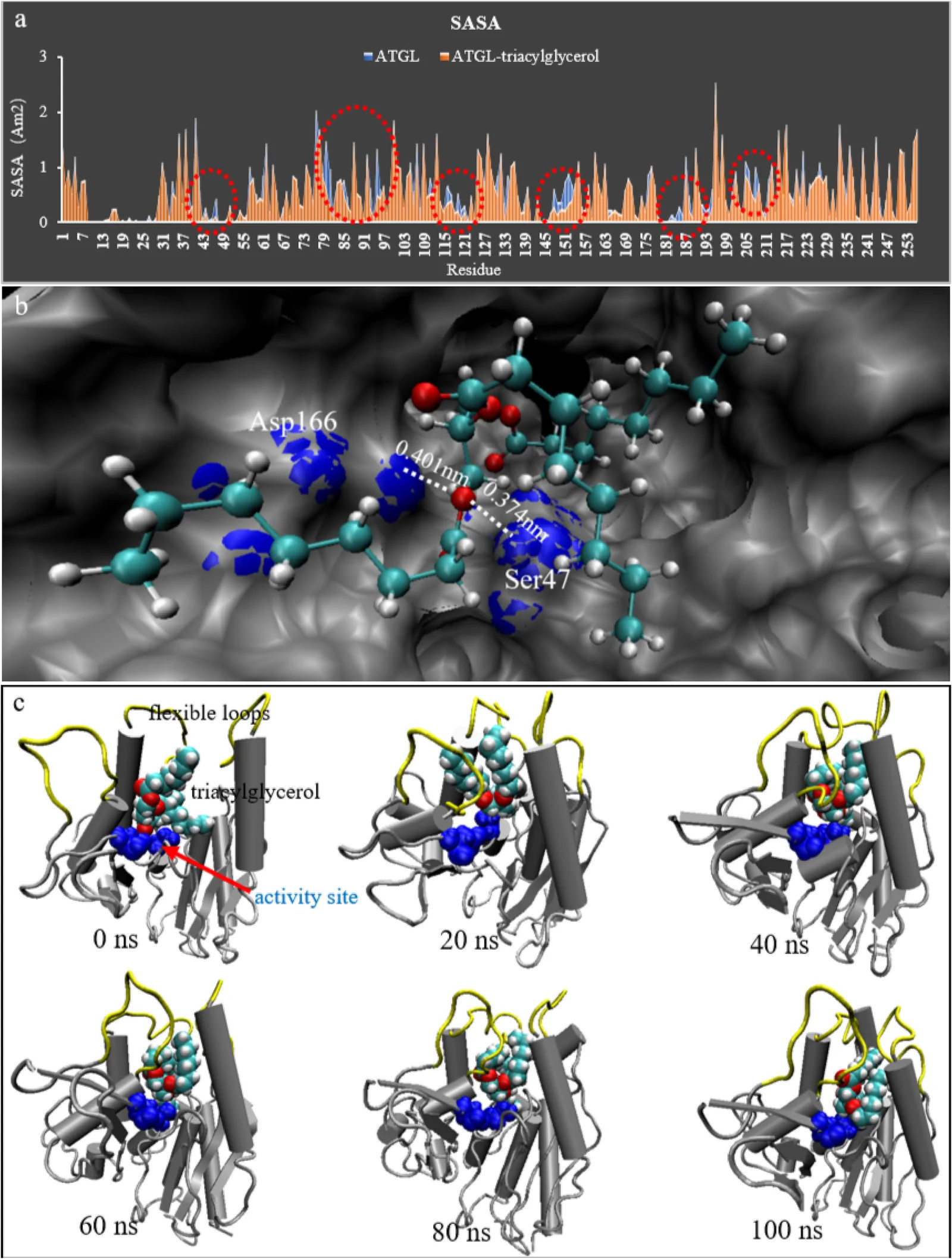
The hydrolysis mechanism of carp ATGL. a: Surface area changes before and after the interaction between ATGL and triacylglycerol; b: Details of the interaction between the ATGL catalytic site and triacylglycerol; c: Conformational changes during the ATGL-triacylglycerol simulation.

Interaction energies between ATGL and the triacylglycerol molecule were calculated using the MM-PBSA method (negative values indicate attraction, positive values repulsion). As shown in Fig. 5a, the total interaction energy was approximately −85.8 kJ/mol, indicating a very stable complex (the bond energy of a carbon–carbon single bond is about 320 kJ/mol). Van der Waals (−118.5 kJ/mol), electrostatic (−11.81 kJ/mol), and hydrophobic interactions (−17 kJ/mol) all favored complex formation, whereas hydrophilic interactions were unfavorable because triacylglycerol molecules are strongly hydrophobic. The negative total energy also indicates that binding of ATGL to triacylglycerol can occur spontaneously in aqueous solution. The contribution of individual amino acid residues to binding was analyzed. Residues such as Phe17, Leu90, Ile148, and His204 (Fig. 5b) are important for ATGL–triacylglycerol binding, and these residues, together with the flexible loops, can serve as targets for inhibitor design.

**Fig. 5:**
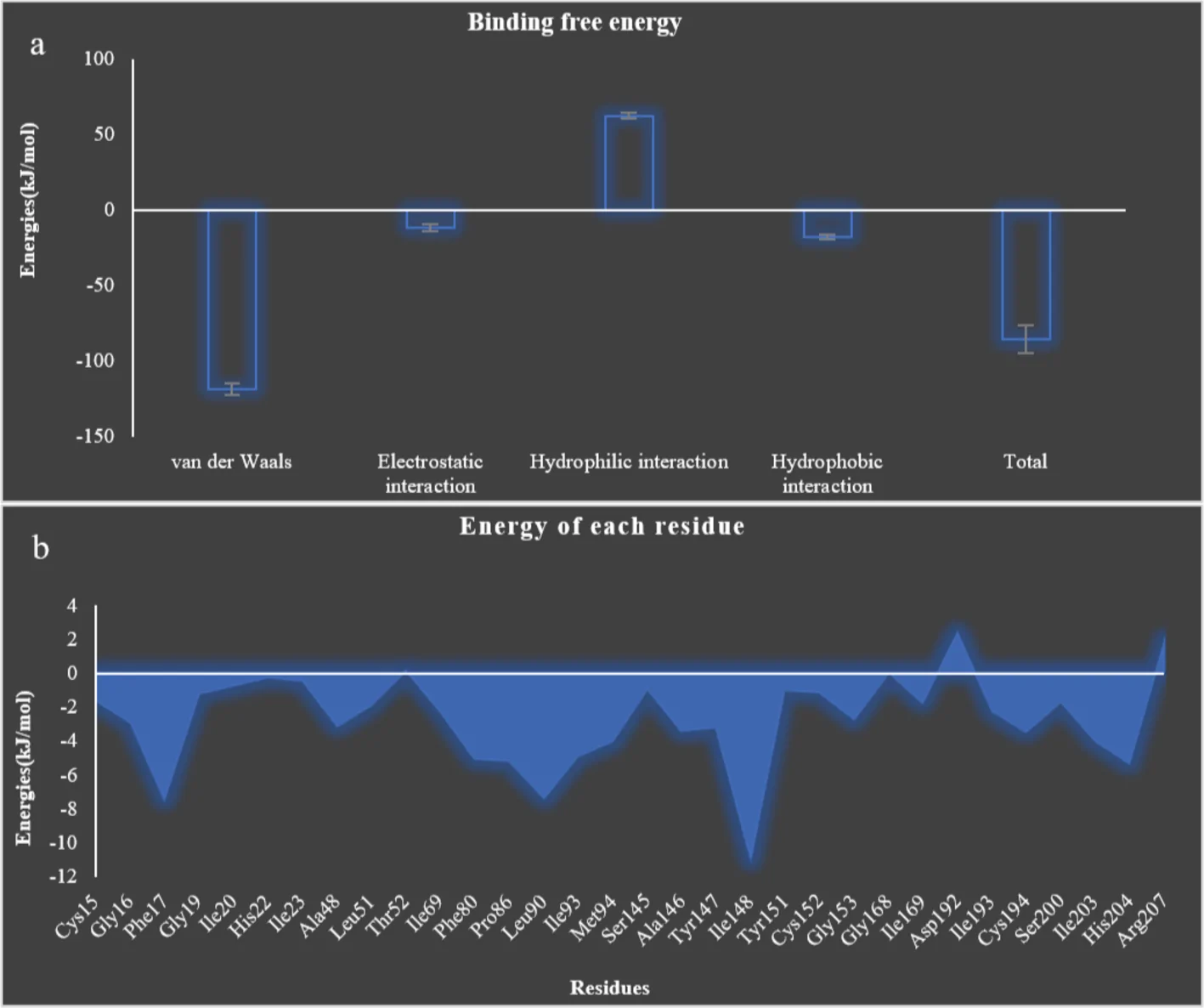
Interaction of ATGL with triacylglycerol molecule a: Binding free energy between ATGL and triacylglycerol; b: Contribution of ATGL amino acid residues to triacylglycerol binding.

### 3.5 Design of an ATGL inhibitor and analysis of the inhibition mechanism

Based on the tertiary structure and catalytic mechanism of ATGL, we designed an efficient inhibitor to enable precise control of carp product quality. Our approach was to use small hydrophobic peptides composed of essential amino acids as competitive inhibitors. First, at sufficient concentration, such peptides can bind to the active groove or hydrophobic loops of ATGL via hydrophobic interactions, creating steric hindrance. Second, peptides made of essential amino acids are safe, non-toxic, and act as nutritional enhancers. Third, they can be produced efficiently and at low cost by genetic engineering (e.g., by designing a cleavage site for enzymatic recycling).

In preliminary experiments, several combinations of hydrophobic essential amino acids were synthesized and tested. The small peptide Lys-Leu-Ile-Met-Val showed the best inhibitory effect on ATGL. HPLC and MS analysis (Fig. 6a,b) confirmed a purity of 95.7% and a molecular weight consistent with the design (approximately 0.6 kDa, pI 9.70). At neutral pH, this small peptide is positively charged and exhibits good solubility. The maximum solubility was approximately 0.7 mg/mL at room temperature and approximately 0.4 mg/mL at 4 °C.

**Fig. 6:**
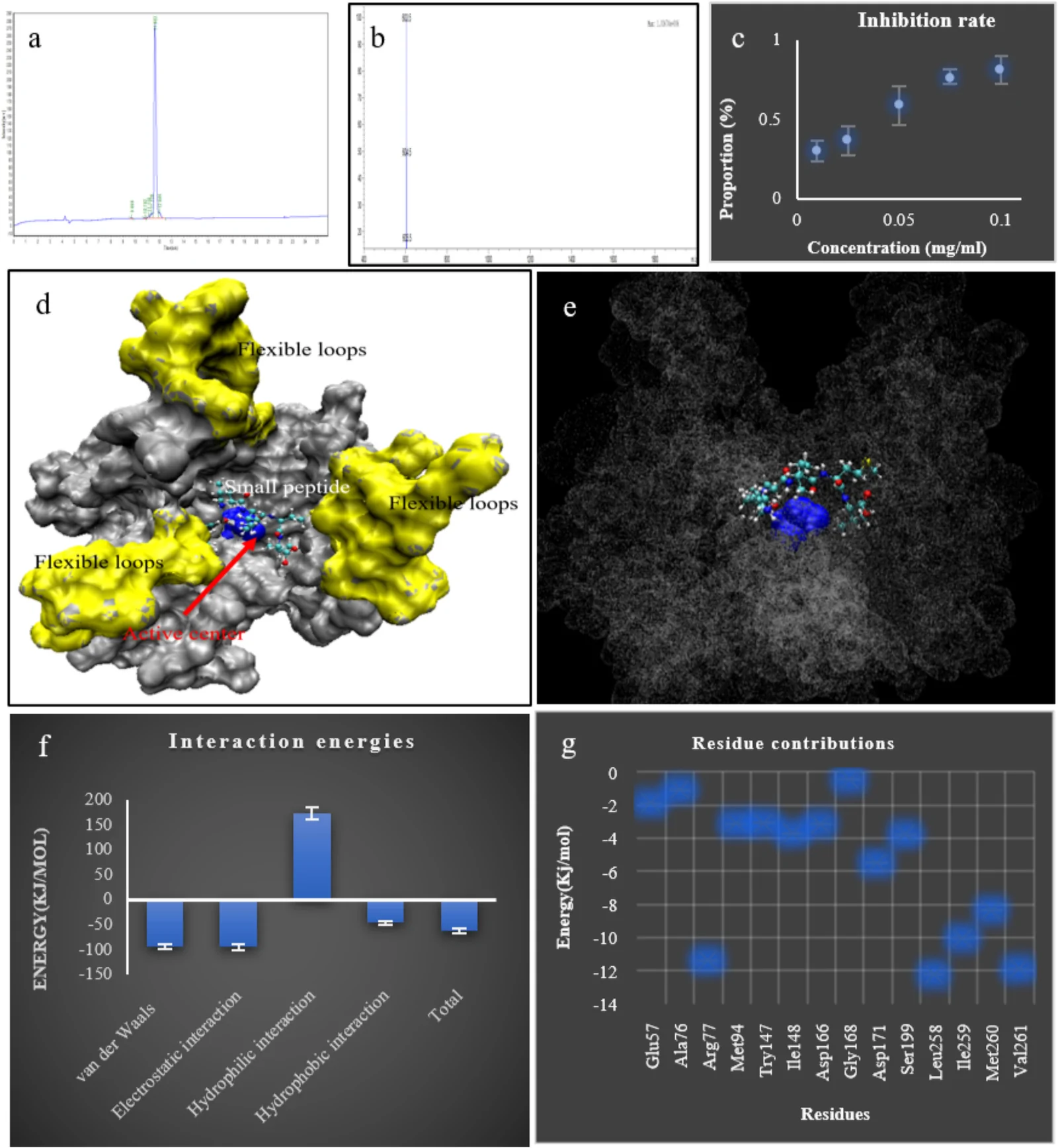
Design of ATGL inhibitors and analysis of the inhibition mechanisms. a: HPLC characterization of ATGL inhibitor. b: MASS characterization of ATGL inhibitor. c: inhibition experiment of carp ATGL. d and e: complexation details of the inhibitor with carp ATGL. f: Binding free energy between ATGL and inhibitor; g: Contribution of ATGL amino acid residues to inhibitor binding.

The peptide was added to the ATGL hydrolysis reaction system (with activity at 25 °C and pH 7 set as 100%). As shown in Fig. 6c, ATGL activity was progressively inhibited with increasing peptide concentration. At 0.1 mg/mL, the hydrolytic activity of ATGL was reduced by 81.5%.

The inhibition mechanism was analyzed by molecular docking and dynamics simulation. Owing to its high hydrophobicity and appropriate size, the small peptide is misrecognized by the hydrophobic active groove of ATGL, forming a steric hindrance that prevents triacylglycerol binding and competitively inhibits ATGL activity (Fig. 6d,e). The total interaction energy between the peptide and ATGL was −62.7 kJ/mol (Fig. 6f), indicating a stable, spontaneous interaction. Residues Arg77, Leu258, and Val261 were found to be particularly important for peptide binding (Fig. 6g).

As shown in Fig. S3, after adding the small peptide to carp surimi and storing at 4 °C for 5 days, the TBARS value and acid value of carp surimi were 8.3% (*P* < 0.05) and 9.9% (*P* < 0.05) lower than those of the control group, respectively. This indicates that the small peptide can effectively control lipid hydrolysis and oxidation in fish flesh, which is closely related to the effective inhibition of ATGL activity by the Lys-Leu-Ile-Met-Val small peptide. In addition, the Lys-Leu-Ile-Met-Val small peptide is almost entirely composed of hydrophobic amino acids. When the small peptide is sufficiently abundant in surimi, whether it may mistakenly bind to endogenous lipoxygenase (LOX) in surimi through hydrophobic interactions, thereby affecting LOX binding to hydrophobic fatty acid substrates, remains to be further verified. Furthermore, the application mode of this small peptide in real fish processing also requires further exploration.

To date, Atglistatin derivatives, G0S2, metformin, and other agents have been found to exhibit significant inhibitory activity against ATGL (Auger et al., 2019; Cerk et al., 2014; Cerk et al., 2018; Gholizadeh et al., 2025; Mayer et al., 2015). G0S2 consists of 103 amino acids. Studies have shown that a 33-amino-acid G0S2 peptide (Lys20-Ala52) lacking both the N- and C-terminal regions also exhibits a potent inhibitory effect on ATGL (Chen et al., 2025; Zhao & Ruan, 2025). Sequence analysis has revealed that this 33-amino-acid G0S2 peptide is highly hydrophobic (Cerk et al., 2014; Gholizadeh et al., 2025). The small peptide designed in the present study is likewise highly hydrophobic. This further indicates that hydrophobic interactions play a predominant role in substrate binding by ATGL. However, metformin, atglistatin derivatives, and similar agents are more likely intended for pharmaceutical use as ATGL inhibitors, rather than being designed for application as food additives.

In addition, the inhibition of spoilage bacteria using agents such as ε-polylysine and PostbioYDFF-3 (Zang et al., 2025; Zhang et al., 2024a; Zhang et al., 2024b), and the suppression of lipid oxidation using rosemary extract, *Ginkgo biloba* extract, and similar antioxidants also represent key strategies for maintaining fish meat quality (Gouvêa et al., 2023; Zeng et al., 2024). Importantly, inhibiting endogenous ATGL activity to control lipolysis can effectively prevent lipid oxidation at the source. The small peptide designed in the present study is composed entirely of essential amino acids; it serves as both an ATGL inhibitor and a nutritional fortifier, and its production cost is relatively low. Therefore, it represents a highly promising additive for carp meat products.

## 4 Conclusion and outlook

Lipid hydrolysis caused by endogenous enzymes is a major factor reducing meat product quality. ATGL initiates triacylglycerol hydrolysis and is thus a key enzyme in this process.

In this study, the N-terminal domain of carp ATGL was cloned from carp muscle tissue. It is 768 bp long, encodes 256 amino acids, has a pI of 7.98, and a molecular weight of 27.7 kDa. The N-terminal domain is highly conserved across species (similarity up to ∼80%). Recombinant ATGL was successfully expressed in *E. coli*, and its activity was characterized at different temperatures and pH values. pH changes had a greater impact on activity, especially under alkaline conditions: when pH increased from 7 to 10, activity decreased by 92.1%. This suggests that adjusting the pH to alkaline values can effectively inhibit ATGL in practice. Temperature had a smaller effect; even at 5 °C or 50 °C, ATGL retained about 73.6% and 62.8% of its activity, respectively, indicating that low-temperature storage alone cannot completely control triacylglycerol hydrolysis.

The predicted tertiary structure of ATGL was analyzed. It is a monomeric protein with nine α-helices and seven β-strands, no disulfide bonds, and an Rg of 1.84 nm. Its active center is a catalytic dyad (Ser47-Asp166) located at the bottom of an active groove approximately 1.9 nm × 1.7 nm that is predominantly hydrophobic. The active groove is surrounded by three flexible hydrophobic loops that are closely involved in binding hydrophobic lipid chains. The hydrolysis mechanism was elucidated: the triacylglycerol molecule becomes embedded in the active groove, with its ester bond contacting the catalytic dyad; the three flexible loops then undergo conformational changes and encapsulate the triacylglycerol, facilitating hydrolysis. The total interaction energy between ATGL and triacylglycerol is approximately −85.8 kJ/mol. Residues such as Phe17, Leu90, Ile148, and His204 are important for this interaction and can serve as targets for inhibitor design.

Finally, an inhibitor of ATGL was designed: a small peptide composed of hydrophobic essential amino acids (Lys-Leu-Ile-Met-Val). At a concentration of 0.1 mg/mL, it reduced ATGL hydrolytic activity by 81.5%. Due to its high hydrophobicity and appropriate size, the peptide is misrecognized by the hydrophobic active groove of ATGL, creating steric hindrance that prevents triacylglycerol binding and competitively inhibits the enzyme. The interaction energy between the peptide and ATGL is −62.7 kJ/mol, indicating stable, spontaneous binding. In addition to its strong inhibitory effect, this essential-amino-acid-derived peptide acts as a nutritional fortifier and has low cost, making it a promising additive for carp meat products. In addition, after the addition of the small peptide, lipid hydrolysis and oxidation in carp surimi during storage were effectively controlled.

Furthermore, in the present study, the analysis of the tertiary structure of ATGL and the molecular details of its interaction with triacylglycerol was primarily based on molecular dynamics simulations. Since the simulation environment (force field, model) may differ to some extent from the actual conditions, these results require further experimental validation. In future studies, techniques such as isothermal titration calorimetry (ITC), surface plasmon resonance (SPR), and small-angle X-ray scattering (SAXS) could be employed to further characterize the structural features of ATGL. Additionally, limited proteolysis coupled with mass spectrometry and site-directed mutagenesis could be applied to investigate whether Ser44-Asp166 and the flexible loop region represent critical sites for substrate binding in ATGL.

This study provides an important reference for basic research on lipid hydrolases and offers a basis for controlling carp meat quality during processing.

## CRediT authorship contribution statement

**Qinghai Wang:** Writing – original draft, Methodology. **Haixia Zhong:** Writing – review & editing, Writing – original draft, Methodology. **Yanhua Jia:** Writing – review & editing, Methodology. **Anhu Wang:** Supervision, Methodology. **Zhiguang Chen:** Writing – review & editing, Writing – original draft, Supervision.

## Ethical statement

Carp tissue collection complied with the Guidelines for Experimental Animals of Xichang University.

## Declaration of competing interest

The authors declare that they have no known competing financial interests or personal relationships that could have appeared to influence the work reported in this paper.

## Data Availability

The authors do not have permission to share data.

## References

[bb0005] Alsted T.J., Nybo L., Schweiger M., Fledelius C., Jacobsen P., Zimmermann R., Kiens B. (2009). Adipose triglyceride lipase in human skeletal muscle is upregulated by exercise training. American Journal of Physiology. Endocrinology and Metabolism.

[bb0010] Auger C., Knuth C.M., Abdullahi A., Samadi O., Parousis A., Jeschke M.G. (2019). Metformin prevents the pathological browning of subcutaneous white adipose tissue. Molecular Metabolism.

[bb0015] Bade P., Stix S., Kappel K., Fritsche J., Haase I., Torda A., Wax N., Fischer M., Brandis D., Schröder U. (2025). A universal DNA microarray for rapid fish species authentication. Food Chemistry: Molecular Sciences.

[bb0020] Cerk I.K., Salzburger B., Boeszermenyi A., Heier C., Pillip C., Romauch M., Oberer M. (2014). A peptide derived from G0/G1 switch gene 2 acts as noncompetitive inhibitor of adipose triglyceride lipase. The Journal of Biological Chemistry.

[bb0025] Cerk I.K., Wechselberger L., Oberer M. (2018). Adipose triglyceride lipase regulation: An overview. Current Protein & Peptide Science.

[bb0030] Chen Y., Johnson S.M., Burr S.D., Povero D., Anderson A.M., McMahon C.E., Liu J. (2025). Absence of the intracellular lipolytic inhibitor G0S2 enhances intravascular triglyceride clearance and abolishes diet-induced hypertriglyceridemia. The Journal of Clinical Investigation.

[bb0035] Cheng Y., Dong J., Zhang H., Zhang Y., Gao F., Wang F., Sun C. (2025). Characterising flavour differences in mandarin fish muscle under live prey and formulated feed diets using flavouromics and metabolomics. Food Chemistry: Molecular Sciences.

[bb0040] Chinese Fisheries (2025).

[bb0045] Eymard S., Carcouët E., Rochet M.J., Dumay J., Chopin C., Genot C. (2015). Development of lipid oxidation during manufacturing of horse mackerel surimi. Journal of the Science of Food and Agriculture.

[bb0050] Gao Y., Fukushima H., Deng S., Jia R., Osako K., Okazaki E. (2018). Effect of pH and heating conditions on the properties of Alaska Pollock (Theragra chalcogramma) surimi gel fortified with fish oil. Journal of Texture Studies.

[bb0055] Gholizadeh M., Asadi J., Aghaei M., Akbar S., Malakouti R., Sepehr K.S., Hesari Z. (2025). The evaluation of PNPLA2, ATGL, and G0S2 levels in serum and PBMCs of the newly diagnosed and the chronic patients with rheumatoid arthritis. International Journal of Rheumatic Diseases.

[bb0060] Gouvêa F.J., de Oliveira V.S., Mariano B.J., Takenaka N.A.R., Gamallo O.D., da Silva Ferreira M., Saldanha T. (2023). Natural antioxidants as strategy to minimize the presence of lipid oxidation products in canned fish: Research progress, current trends and future perspectives. Food Research International.

[bb0065] Han G., Zhang L., Li Q., Wang Y., Chen Q., Kong B. (2020). Impacts of different altitudes and natural drying times on lipolysis, lipid oxidation and flavour profile of traditional Tibetan yak jerky. Meat Science.

[bb0070] Han S.L., Liu Y., Limbu S.M., Chen L.Q., Zhang M.L., Du Z.Y. (2021). The reduction of lipid-sourced energy production caused by ATGL inhibition cannot be compensated by activation of HSL, autophagy, and utilization of other nutrients in fish. Fish Physiology and Biochemistry.

[bb0075] Hu X., Zeng Z., Zhang J., Wu D., Li H., Geng F. (2023). Molecular dynamics simulation of the interaction of food proteins with small molecules. Food Chemistry.

[bb0080] Kamal H., Ali A., Manickam S., Le C.F. (2023). Impact of cavitation on the structure and functional quality of extracted protein from food sources – An overview. Food Chemistry.

[bb0085] Kulminskaya N., Oberer M. (2020). Protein-protein interactions regulate the activity of adipose triglyceride lipase in intracellular lipolysis. Biochimie.

[bb0090] Li Y., Dong X., Zhang Y., Xiao T., Zhao Y., Wang H. (2023). Astragalus polysaccharide improves the growth, meat quality, antioxidant capacity and bacterial resistance of Furong crucian carp (Furong carp × red crucian carp). International Journal of Biological Macromolecules.

[bb0095] Li X., Chen J., Li J., Zhang Y., Xia J., Du H., Sheng C., Huang M., Shen W., Cai G., Wu L., Bai X., Chen X. (2025). ATGL regulates renal fibrosis by reprogramming lipid metabolism during the transition from AKI to CKD. Molecular Therapy.

[bb0100] Liu D.M. (2013).

[bb0105] Marzouk Y., Zaki M.A., Nour A.M., Mehrim A.I., Khalil H.S. (2025). Impacts of commercial bile acids on growth performance, immune responses and expression genes of lipid metabolism in Nile tilapia fingerlings *Oreochromis niloticus*. Scientific Reports.

[bb0110] Mayer N., Schweiger M., Melcher M.-C., Fledelius C., Zechner R., Zimmermann R., Breinbauer R. (2015). Structure–activity studies in the development of a hydrazone based inhibitor of adipose-triglyceride lipase (ATGL). Bioorganic & Medicinal Chemistry.

[bb0115] Nagy H.M., Paar M., Heier C., Moustafa T., Hofer P., Haemmerle G., Zimmermann R. (2014). Adipose triglyceride lipase activity is inhibited by long-chain acyl-coenzyme a. Biochimica et Biophysica Acta.

[bb0120] Pulinilkunnil T., Kienesberger P.C., Nagendran J., Sharma N., Young M.E., Dyck J.R.B. (2014). Cardiac-specific adipose triglyceride lipase overexpression protects from cardiac steatosis and dilated cardiomyopathy following diet-induced obesity. International Journal of Obesity.

[bb0125] Recazens E., Mouisel E., Langin D. (2021). Hormone-sensitive lipase: Sixty years later. Progress in Lipid Research.

[bb0130] Schreiber R., Xie H., Schreiber M. (2019). Of mice and men: The physiological role of adipose triglyceride lipase (ATGL). Biochimica et Biophysica Acta - Molecular and Cell Biology of Lipids.

[bb0135] Schweiger M., Eichmann T.O., Taschler U., Zimmermann R., Zechner R., Lass A. (2014). Measurement of lipolysis. Methods in Enzymology.

[bb0140] Sobczak M., Panicz R., Eljasik P., Sadowski J., Torz A., Żochowska-Kujawska J., Dias J. (2020). Quality improvement of common carp (*Cyprinus carpio* L.) meat fortified with n-3 PUFA. Food and Chemical Toxicology.

[bb0145] Tang T., Abbott M.J., Ahmadian M., Lopes A.B., Wang Y., Sul H.S. (2013). Desnutrin/ATGL activates PPARδ to promote mitochondrial function for insulin secretion in islet β cells. Cell Metabolism.

[bb0150] Wang J., Qian Q., Ren L.Q. (2020). Study on the changes in fish quality of carp under frozen storage. Quality and Safety of Agricultural Products..

[bb0155] Zanfirescu A., Ungurianu A., Mihai D.P., Radulescu D., Nitulescu G.M. (2021). Targeting monoacylglycerol lipase in pursuit of therapies for neurological and neurodegenerative diseases. Molecules.

[bb0160] Zang J., Li T., Liu K., Wu J., Zhang Z., Liu X., Li Z. (2025). Correlations between microbiota succession and volatile profiles development and biogenic amine formation involved in the ripening of Chinese sour meat. LWT.

[bb0165] Zeng J., Song Y., Fan X., Luo J., Song J., Xu J., Xue C. (2024). Effect of lipid oxidation on quality attributes and control technologies in dried aquatic animal products: A critical review. Critical Reviews in Food Science and Nutrition.

[bb0170] Zhang Z., Zhao J., Zang J., Peng C., Lv L., Li Z. (2024). The inhibition mechanism of PostbioYDFF-3 on quality deterioration of refrigerated grass carp fillets from the perspective of endogenous enzyme and microorganisms changes. Food Chemistry.

[bb0175] Zhang Z., Zhao J., Zang J., Peng C., Lv L., Li Z. (2024). The enhanced antibacterial effect and mechanism of PostbioYDFF-3 against pseudomonas putida NBRC 14164 and Acinetobacter johnsonii ANC 3681. Food Bioscience.

[bb0180] Zhao X.H., Ruan L. (2025). Recent advances on the role of G0S2. Discover Oncology.

[bb0185] Zheng Y., Chen J., Macwan V., Dixon C.L., Li X., Liu S., Neculai D. (2024). S-acylation of ATGL is required for lipid droplet homoeostasis in hepatocytes. Nature Metabolism.

[bb0190] Zhong H.X., Chen Z.G., Lin J.H., Xu Y., Liu D., Yin X.F. (2021). The inhibition mechanism of carp (*Cyprinus carpio*) stefin to cathepsin B and their tertiary structures. International Journal of Food Science and Technology.

[bb0195] Zhong H.X., She Y.X., Yang X.J., Wen Q., Chen L., Wang X.B., Chen Z.G. (2024). Analysis of the interaction between double-helix starch molecule and α-amylase. Innovative Food Science and Emerging Technologies.

[bb0200] Zhong H.X., She Y.X., Yang X.J., Wen Q., Chen L., Wang X.B., Chen Z.G. (2024). Analysis of the mechanism of resistance to enzymatic hydrolysis of RS-5 resistant starch. Food Chemistry.

