# Supplementary material for: Cloning, expression, tertiary structure, activity characteristics, design of inhibitor, hydrolysis and inhibition mechanisms analysis of carp adipose triglyceride lipase (ATGL)
Source: Food Chem (Oxf). 2026 Sep 30;13:100460. doi: 10.1016/j.fochms.2026.100460 (PMC13659687; doi:10.1016/j.fochms.2026.100460)
Supplement: Supplementary file 1 [file mmc1.docx]

Fig. S1 carp ATGL primary structure. a: Carp ATGL primary structure, b: RNA from carp muscle tissue, c: Carp ATGL PCR amplification, d: Carp ATGL DNA and amino acid sequences

Fig. S2 Amino acid sequence alignment

Fig. S3 Effect of the small peptide on the TBARS value and acid value of minced carp muscle
